# Maximizing benefits from evidence-based psychological treatments: Memory support and habit formation as key strategies

**DOI:** 10.1016/j.brat.2025.104767

**Published:** 2025-05-13

**Authors:** Allison G. Harvey

**Affiliations:** University of California, Berkeley, CA, USA

**Keywords:** Memory support intervention, Habit formation, Evidence-based psychological treatments, Behavior change, Experimental therapeutics approach, Text messages, Cognitive behavior therapy, Parenting

## Abstract

Evidence-based psychological treatments (EBPTs) aim to reverse psychological processes that contribute to the development and/or maintenance of mental illness. Developed and rigorously tested through scientific research, EBPTs effectively address a broad range of mental health challenges, often as front-line treatments. However, there is potential to further improve outcomes. This paper examines two strategies for maximizing the benefits of EBPTs. The first addresses the concerning and well replicated finding that patients accurately recall only about one-third of the treatment points discussed during a session. This poor memory for treatment negatively impacts adherence to the EBPT and outcomes from the EBPT. The process of developing and testing the Memory Support Intervention (MSI), to improve patient memory for treatment, is described. The latter involved leveraging findings from cognitive psychology and education to develop memory support strategies to add to EBPTs, with the goal of improving EBPT outcomes. The second strategy for maximizing the benefits of EBPTs highlights the potential of habit formation principles to enhance EBPTs. While a core goal of EBPTs is to reduce unhelpful habits and encourage adaptive ones, the science of habit formation has not been fully integrated. The Habit-based Intervention (HABITs) was developed to explicitly incorporate habit formation elements into EBPTs. Both the MSI and HABITs are designed as adjunctive interventions, enhancing EBPTs without increasing the number or duration of sessions. This paper concludes by emphasizing the importance of leveraging insights from diverse fields of basic science to uncover new strategies for improving both the short- and long-term outcomes of EBPTs.

I recently participated in an evidence-based psychological treatment (EBPT) as a patient. My goal was to improve my parenting skills. I participated in 11 weekly, 1 h group sessions, which were lively, interesting and surprisingly fun. The two clinical psychologists who ran the group were excellent. They were wise, knowledgeable, organized and warm. I felt proud to see my profession shining so brightly! The 1-h sessions were divided in half, with about 30 min devoted to reviewing the homework for the prior week, while the remaining 30 min was devoted to new content. While I am not trained to deliver parent training interventions, I sensed that this was a truly excellent “dose” of this EBPT.

I was a highly motivated patient. Every week I took notes during the session. After the session, I printed out the handouts and I completed the homework studiously. Also, I actively participated in every group by asking questions and volunteering the progress I had made. Yet, at the end of the 11th session I was surprised to have forgotten much of the content of the treatment. Also, my new parenting skills were far from habitual. Looking back, I regret that each week of new content was only practiced for a mere 7 days before the next new content was presented. And, at least once, the 7 days included some atypical aspect (e.g., I was travelling) that made it impossible to try out the new skill. My take-home from this experience is that there’s little chance that even the most motivated patient can maximally benefit from EBPTs structured like parent training because of two implicit assumptions. First, it was assumed that the parent had successfully encoded long-term memories of the new content introduced in only one session and reviewed in only one session. Second, it was assumed that each new parenting skill had become habitual with 7 days of practice. Although my team and I have been researching these two assumptions, that are inherent to many EBPTs, this experience as a patient served as a powerful reminder of the need for more research to explore methods to maximize the short- and long-term benefits from EBPTs.

This paper describes the process—along with the surprises, disappointments, and challenges—of two approaches my team at the University of California, Berkeley, has been exploring to augment EBPTs. It is important to acknowledge that the general approach we have taken follows a well-trodden path paved by many esteemed pioneers of our field, whose contributions to improving EBPTs have leveraged a “mixture of phenomenological, experimental and treatment development studies” (p. 1089) (Clark, 2004). Building on this tradition, an excellent meta-analysis by Nord et al. (2023) underscored the value of investigating ways to augment EBPTs. Their focus was “augmentations that are delivered before, during or after a session of psychological therapy” with the intention of improving outcome (p. 389). The augmentations covered included using imagery to enhance the impact of a particular treatment point and bias modification to alter the cognitive bias to threat that may play a causal role in maintaining psychological distress. Nord et al. (2023) concluded that augmentations have a meaningful positive impact on outcomes, with effect sizes ranging from small to moderate. They also highlighted additional benefits, such as a low risk of adverse effects and the potential for improved outcomes at minimal additional cost.

## 1. Patient memory for treatment

Consistent with my own experience of participating in an EBPT, multiple studies show that patients accurately recall only about *one-third* of the recommendations made during a doctor’s visit (e.g., Bober et al., 2007; Croyle et al., 2006; Jansen et al., 2008; Lewkovich & Haneline, 2005; Ley, 1979; Mcguire, 1996; Pickney & Arnason, 2005) and during a session of cognitive behavior therapy (CBT) (e.g., Chambers, 1991; Gumport et al., 2018; Hahlweg & Richter, 2010; Lee & Harvey, 2015). Furthermore, in one study, 25% of patients remembered recommendations that were never actually made (Bober et al., 2007). This poor memory for the content of treatment is associated with poor adherence and worse outcome (Dong et al., 2017a; Kravitz et al., 1993; Lee & Harvey, 2015; Ley et al., 1977; Sarfan, Zieve, Mujir, et al., 2023; Zieve et al., 2020).

While shocking at one level, these findings are perhaps not surprising when we consider that (1) even when memory is functioning optimally, fallibility is possible at initial encoding, storage and retrieval (Schacter, 2001), (2) EBPT treatment sessions are lengthy (e.g., 50 min), involve complex information, and may evoke negative emotions, which can narrow attention and subsequently impair encoding (Easterbrook, 1959) and (3) people’s daily lives are often filled with activities and concerns that can overshadow, distract from, or compete with their ability to recall the content of treatment.

Inspired by the emerging data, my team and I set out to develop a method for enhancing patient memory for treatment. Our research journey has been filled with challenges and unforeseen complexities, and there’s still much work ahead. Let me walk you through the steps we took.

We began by reviewing the vast cognitive science and education literatures. This resulted in a (very) long list of evidence-based memory supports. From these, we used the four criteria listed in Table 1 to guide the selection of 8 memory supports presented in Table 2 (also see Table 1 in Sarfan, Zieve, Gumport, et al., 2023 for examples of each type of memory support). We published this process and the findings in *Perspectives in Psychological Science* (Harvey et al., 2014).

We were aware that many EBPTs already have at least some memory support built in. For example, CBT involves memory supports such as capsule summaries, note taking of major points, writing down the homework list, recall of the prior session at the beginning of each session and a recap at the end of each session (e.g., Beck, 1995; Beck, 2005). However, at the time, we didn’t know the amount of memory support, nor the type of memory support delivered in treatment-as-usual and we didn’t know if raising the quality and amount of memory support would improve patient memory and the overall outcome from a course of therapy.

### 1.1. Pilot study testing the Memory Support Intervention (MSI)

We then designed and conducted a pilot study in which we trained our therapists to integrate the 8 memory supports proactively and strategically into treatment-as-usual to support patients to fully encode the content of the treatment. The therapists were trained to first identify each ‘treatment point’ they made. A treatment point was defined as an insight, skill, or strategy that is part of the treatment (Lee & Harvey, 2015). Once a treatment point was identified, therapists delivered one or more of the 8 memory supports (listed in Table 2).

As an initial “platform” for investigating the MSI, we recruited patients who met diagnostic criteria for major depressive disorder and we focused on one EBPT—cognitive therapy (CT). We compared CT for depression plus memory support (CT + MSI; n = 25) to CT for depression-as-usual (CT-as-usual; n = 23) for adults 18 years of age and older (Dong et al., 2017b; Gumport et al., 2018; Harvey et al., 2016). The initial step for the data analysis was to assess whether the MSI enhanced memory support in the CT + MSI group compared to the CT-as-usual group. The findings indicated that the MSI successfully increased the use of memory support strategies. Specifically, therapists in the CT + MSI group provided an average of 18 memory supports, while those in the CT-as-usual group delivered an average of 8. Next, we found that individuals in the CT + MSI group reported more accurate thoughts and applications of treatment points at each assessment, relative to CT-as-usual. Though not statistically significant, the CT + MSI group exhibited greater reduction in depression severity from before to after the treatment, and the odds of meeting criteria for ‘response’ and ‘remission’ were higher in CT + MSI compared with CT-as-usual. CT + MSI also showed an advantage on functional impairment. Interestingly, greater treatment effects were observed for those who had less than 16 years of education. Given the small size and limited power of this study, we viewed these initially promising results as preliminary at best.

### 1.2. A second test of the MSI

Our next step was to conduct a confirmatory efficacy trial of the MSI, continuing to deliver the 8 memory supports. Using the same platform of major depressive disorder and CT, we randomly allocated 178 adults aged 18 years of age and older to either CT + MSI (n = 91) or to CT-as-usual (n = 87) (Dong et al., 2022). We were initially disappointed to learn that although the mean values were in the expected direction, the CT + MSI group was essentially equal to the CT-as-usual group with only three exceptions. At the 6-month follow-up, CT + MSI were less depressed compared to CT-as-usual. Also, although not significant, there was a small effect size difference in the rate of relapse, favoring the CT + MSI group (26 %), relative to the CT-as-usual group (42 %). Finally, CT + MSI was superior to CT-as-usual on one of the two functional impairment measures; namely, the index of unhealthy days from the CDC Healthy Days Measure, such that the CT + MSI group showed greater reduction in unhealthy days from pre-treatment to 6-month follow-up. It’s notable that the effects of memory support appear to be most evident after the 6-month follow-up not at the post-treatment assessment. Interestingly, the results for education in the pilot did not replicate. In fact, we found the opposite result! Taken together, we were confused and disappointed.

**Fig. 1.**
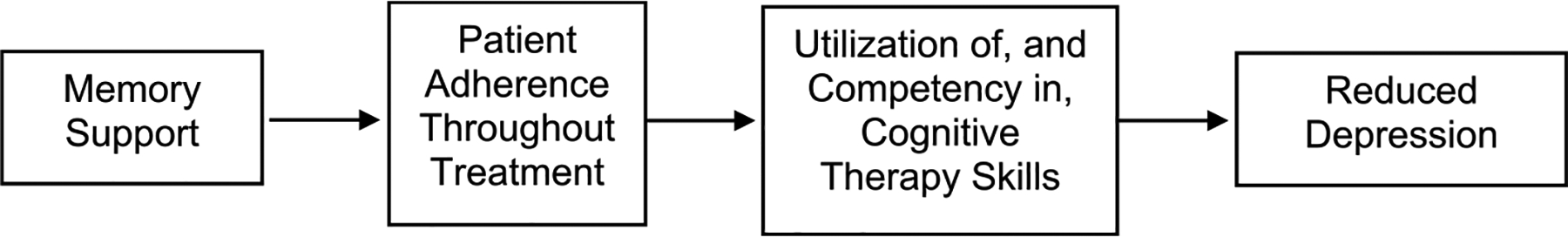
Memory Support Increases Adherence which, in turn, Increases Skills and Competency, Leading to Improved Depression Outcome (Sarfan, Zieve, Gumport, et al., 2023).

During the inevitable “postmortem”, several new insights and directions emerged. First, there’s the very practical consideration that CT-as-usual is an excellent treatment and may be hard to substantively improve upon. Second, the therapist’s memory for the content of treatment was assessed at sessions 4, 8, 12 and 16. Informally, due to these assessments, several CT-as-usual therapists reported that they deduced the study was about memory for treatment. It is possible that these therapists started delivering idiographic versions of memory support. Third, Dr. Garret Zieve, who was a graduate student at the time, suggested that some of the 8 memory support strategies may more effectively increase patient memory for treatment than others. He started researching memory support strategies that incorporate *constructive learning*—defined as strategies that provoke inferences beyond the information presented (Chi et al., 2001; Chi & Wylie, 2014; Menekse et al., 2013). Dr. Zieve proposed that 4 of the original 8 memory supports were “constructive” in that they prompted learners to generate new ideas, inferences or connections that go beyond what is explicitly presented (see Table 2). Using data from the pilot study described above, he also showed that these constructive memory supports resulted in better outcomes, compared to non-constructive strategies (Zieve, Dong, et al., 2019). Then Dr. Laurel Sarfan, a post-doctoral scholar on our team at the time, tested whether the dose of constructive memory support that was delivered in CT + MSI (an average of 5 per session) was sufficient in the efficacy trial described above. Dr. Sarfan discovered that we should have delivered about 8 constructive strategies per session to optimize all patient recall, outcomes, and mechanisms of memory support strategies (Sarfan, Zieve, Gumport, et al., 2023). The benchmark for these analyses was to find the dose that would equate to a 50% probability of achieving clinically meaningful change. Fourth, Dr. Sarfan published another creative and important paper in which she showed that the relationship between memory support and patient outcome was more nuanced than a simple direct effect. Specifically, as depicted in Fig. 1, she found that memory support strategies appear to operate indirectly through mechanisms such as improved patient adherence to treatment, more use of CT skills, and greater competency in CT skills (Sarfan, Zieve, Mujir, et al., 2023). Fifth, Dr. Zieve had the idea to conduct a deep dive into the data generated from our second test of the MSI because he was intrigued by the possibility of investigating opportunities to improve the efficacy of the MSI. He discovered that too many memory support strategies were targeted towards memory for the basic concepts of CT (e.g., the CBT model) as opposed to the practical applications (e.g., change strategies) (Zieve et al., 2023). Based on this data, we realized we should have trained the therapists to spread their use of memory support across treatment contents and to ensure a strong focus of memory support on procedural treatment contents (e.g., practical intervention recommendations) rather than on the conceptual treatment contents (e.g., the underlying theoretical model used in treatment) (de Jong & Ferguson-Hessler, 1996).

Taken together, the innovative ideas from various team members motivated us to test a new version of the MSI, focused solely on the four constructive memory supports. In this new study, we are also ensuring that the lessons we learned from the efficacy trial are incorporated and we have introduced new methods to ensure, and measure, the quality of the memory support provided (Milner et al., 2024). Additionally, this research focuses on older adults, whom we previously hypothesized to be at higher risk for poorer outcomes due to challenges with memory for treatment (Harvey et al., 2014). As older adults tend to use more health services (Zayas et al., 2016) and healthy aging is associated with declines in memory functioning (Glisky, 2007; Li et al., 2016; Parikh et al., 2016; Salthouse, 2009; Ward et al., 2020), boosting memory for treatment may be particularly impactful. We eagerly await the results from this study!

### 1.3. Preparing for deployment

Research on human memory has been a rich and long endeavor in psychology (e.g., Baddeley, 2014; Ebbinghaus, 2013; Tulving, 1989). There are countless ways to improve memory that are likely to be relevant to the challenge of improving patient memory for treatment. We selected the MSI approach with a laser-like focus on maximizing the chance that the MSI will be feasible for therapists within routine practice (Onken et al., 2014; Weisz et al., 2014). For example, we selected memory supports that can plausibly be used within typical EBPT treatment sessions, and we ensured that the intervention does not add to session length or to treatment duration. Furthermore, during her time as a graduate student on our team, Dr. Niki Gumport developed and initiated the validation process for a provider-rated checklist designed to assess memory support. Our rationale was that the “gold standard” research method for assessing fidelity to the MSI—requiring trained independent raters to review session recordings (Lee et al., 2016)—is overly burdensome and impractical in routine mental health care settings. Hence, the provider-rated measure of the use of memory support offers a viable, brief method to evaluate fidelity to the MSI (Gumport et al., 2021). Additionally, our team surveyed mental health practitioners regarding their use of, and the perceived utility of, the MSI. We concluded that strategies aimed at enhancing patient memory for the content of treatment are likely to be well-received by practicing therapists (Zieve, Gumport, et al., 2019). Overall, focusing on preparing to deploy new approaches—such as the MSI—is a crucial task from the outset for all of us who seek to develop and improve treatments (Onken et al., 2014; Weisz et al., 2014).

### 1.4. Future directions

There is still a great deal to accomplish, with many promising applications of the MSI that merit further research. This section shares ideas about some key avenues worth exploring.

#### 1.4.1. Does therapist memory for treatment matter?

As a therapist, how confident are you in your ability to accurately recall the treatment sessions you provide? As part of the confirmatory efficacy trial described above, the amount of memory support delivered and memory for treatment points by the therapists was measured at the end of sessions 2, 4, 8, and 12, and at the final session. Three types of memory support (non-constructive, constructive, and overall) were analyzed. Led by Catherine Callaway, a graduate student currently on my team, the results were intriguing. The therapists who were delivering CT + MSI recalled more treatment contents compared to CT-as-usual. However, to our surprise—and opposite to the patient finding—delivering *non-constructive* MS increased therapist recall more than delivering constructive MS (Callaway, Zieve, & Harvey, 2025). We interpreted this finding as suggesting that delivering non-constructive memory support might actually serve as constructive MS for the therapist, as it requires the therapist to “do the work” (e.g., the therapist needs to recall and repeat a therapy point). In contrast, delivering constructive MS may be non-constructive for the therapist, as it shifts the responsibility to the patient to “do the work” (e.g., the therapist simply asks a memory support question such as: “Of all these strategies, which would you say is most helpful for managing rumination?” prompting the patient to recall and evaluate the different strategies). Also, better therapist recall was associated with significant improvement on several patient outcomes from pre-treatment to post-treatment and on to 6-month follow-up, but not at 12-month follow-up. In other words, therapist memory for treatment did improve patient selected outcomes short-term (post--treatment, 6-month follow-ups) but not longer term (12-month follow-up) (Callaway, Milner, Zieve, Ashlock, & Harvey, 2025)). Together, these findings underscore the potential significance of therapist memory for treatment content and raise an empirical question for future research, namely: could enhancing therapist memory for treatment lead to improved outcomes?

#### 1.4.2. Boosting patient memory for treatment after the initial course of treatment is completed

So far, our exploration in this area has been preliminary. Let me first provide some background on the main study to which we added memory supports six months after the completion of the treatment.

We sought to improve sleep and circadian functioning among 176 youth who were 10–18 years of age. We compared the Transdiagnostic Sleep and Circadian Intervention (TranS-C) (Harvey & Buysse, 2017) with psychoeducation (PE). Relative to PE, TranS-C was associated with an improvement in selected sleep, circadian and health outcomes (Harvey et al., 2018). Between the 6 and 12-month follow-up, we attempted to improve patient memory for treatment by sending text message reminders of the content of treatment. Specifically, participants were randomized to receive “PUSH” text messages that repeated treatment information (n = 47), “PULL” text messages that prompted the recall of treatment information (n = 50), or no text messages (n = 47). Based on evidence from the cognitive science literature that practice remembering may be more effective than repetition for memory encoding (Karpicke & Roediger, 2007), we predicted that the PULL texts which prompt practice remembering would be more effective than the PUSH texts which constitute repetition. The results indicated that TranS-C treatment effects, relative to PE, were enhanced among participants who received text messages relative to those who did not receive the texts. Although there were no differences between the “PUSH” and the “PULL” texts, the direction of the mean values were in the predicted direction. Overall, insufficient sample size (20–25 participants per cell) precluded a clear conclusion, except that this is a promising domain for future research.

#### 1.4.3. Between session memory support

Thew (submitted) has emphasized the importance of memory as a key between-session process that can influence engagement and treatment outcome. More specifically, research is needed to explore whether—and which—memory support strategies enhance the encoding of treatment content between therapy sessions and how this influences adherence to homework and treatment outcome. One promising approach that could be adapted for this purpose is Hallford’s (2024) self-guided recall homework task.

#### 1.4.4. Digital technology

Delivering EBPTs through digital technology has been a huge growth area and presents an excellent opportunity to improve access to mental health services. However, one potential challenge of digitally delivered interventions is retaining patient engagement. Unlike in-person interactions, digital platforms often lack the personal touch and accountability that may help maintain consistent engagement. Patients can lose motivation or become distracted by other online activities.

Saleem et al. (2021) reviewed the engagement strategies currently used in digital interventions. These authors concluded that existing effective approaches to improving intervention outcomes include providing personalized support during the intervention, facilitating access to social support, and offering tailored feedback. Could memory support represent another meaningful addition to the engagement toolkit for digital versions of EBPTs? This possibility raises several compelling questions: To what extent do patients retain the key treatment points presented during digital interventions? Can memory supports be integrated into these interventions to bolster patient recall of critical treatment information? And, if so, how might enhanced memory recall translate into improved adherence to recommended actions, ultimately leading to better treatment outcomes? Exploring these questions could help unlock new strategies for maximizing the effectiveness of digital health interventions.

#### 1.4.5. Who else might benefit?

Certain patient populations may derive particular benefit from incorporating memory support into EBPTs. For example, given that memory systems continue to develop throughout childhood (Ghetti & Bunge, 2012), is there an opportunity to augment outcomes of treatments for children? For example, might outcomes from CBT for children diagnosed with an anxiety disorder, such as Coping Cat (Kendall & Hedtke, 2006), improve by adding memory support? As another example, ADHD is associated with difficulties across various dimensions of memory (Fosco et al., 2020). CBT for ADHD involves strategies and skills to manage ADHD symptoms like inattention, along with skills in organization and time management (Safren et al., 2010). To what extent does CBT for ADHD already include memory support and could adding more memory support improve outcome? Relatedly, is it possible that specific types of memory support are more effective for people who have ADHD? These are exciting questions for future research.

### 1.5. Summary

**Fig. 2.**
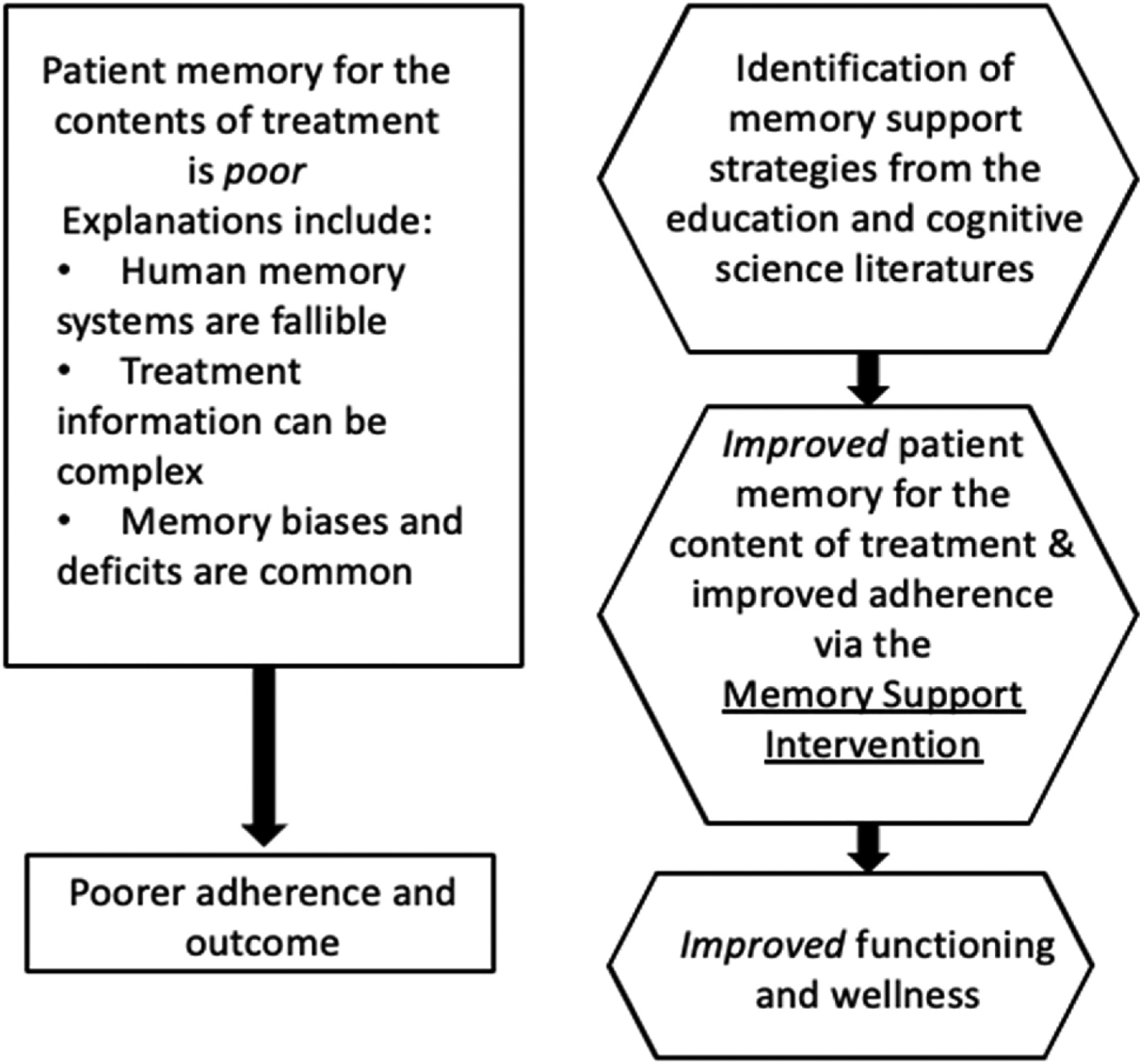
Conceptual framework depicting memory impairment as a pathway to poorer outcomes (rectangles) and the memory support intervention as a pathway to improved outcome (hexagons).

As summarized in Fig. 2, this research program arises from the observation that patient memory for the content of treatment is poor and this contributes to poorer adherence and poorer outcome. The MSI was derived from the education and cognitive science literatures to improve patient memory for the content of treatment and improve adherence, with positive knock-on effects for improving patient functioning. This has been a scientific and creative endeavor with a mix of disappointing results that triggered deeper exploration and then several exciting discoveries. This program of research demonstrates the immense value of team science and that the path from “bench to bedside” is not linear!

## 2. Habit formation

In the parenting EBPT I recently completed, there appeared to be an assumption that practicing a new skill for just 7 days is enough to establish a new parenting habit and break an unhelpful one. However, despite my best efforts, 7 days wasn’t even close to sufficient for either building lasting new habits or breaking old, unhelpful habits. Here it’s important to recognize that EBPTs take various forms. The parenting intervention I received was delivered in a group setting with pre-specified content for each session. However, EBPTs at the opposite end of the continuum—where the content is highly individualized and guided by ongoing case formulation—also seem to carry an implicit expectation that habit formation will somehow *just happen*. Indeed, the process of habit formation in EBPTs tends to be a “passive phenomenon,” or assumed to be “a ‘natural’ outcome of the behavior change process” (Stokes & Baer, 1977, p. 349). However, in reality, habit formation is a process that can be specifically planned for, guided and measured (Gardner et al., 2012).

My team has become interested in exploring how to more directly integrate the science of habit formation into EBPTs to augment longer-term outcomes (Harvey et al., 2022). Habit formation is a learned process whereby a behavior (the desired habit) becomes paired with a stable context cue and, via repetition, the cue eventually triggers an automatic impulse to engage in the habit (Gardner, 2015; Verplanken, 2018). To me, this process should be an *explicit* goal of EBPTs. The following sections will outline components of the habit formation process that could be integrated into EBPTs. I demonstrate their application in the parenting EBPT as an example of how they could be used in any EBPT.

### 2.1. Education

In the parent training EBPT, I would have benefited from explicit education on habit formation and disruption processes, perhaps following the approach taken in rumination-focused CBT (RFCBT) (Watkins, 2018). In RFCBT, the therapist explains the characteristics of rumination as a habit. This explanation includes that rumination tends to be automatic, that rumination is triggered by various cues and that rumination will be hard to change and will recur under conditions of stress or tiredness. RFCBT also prepares patients for inevitable setbacks in breaking the rumination habit. In the same way, within parent training, it would have been beneficial to highlight that unhelpful parenting habits are often automatic and difficult to change, especially when the parent is stressed and tired. If parents understand the science of habit formation and anticipate these challenges, they may be more likely to persist and renew their efforts when old patterns resurface, rather than feeling discouraged and giving up.

### 2.2. Repetition

Research in health psychology indicates that the process of forming habits can take anywhere from 18 days to 36 weeks (e.g., Fournier, d’Arripe-Longueville et al., 2017; Fournier, d’Arripe-Longueville et al., 2017; Kaushal & Rhodes, 2015; Lally et al., 2010). These studies typically involve one relatively discrete habit like increasing physical activity (Fournier, d’Arripe-Longueville et al., 2017) or eating healthy foods (Lally et al., 2010). Considering this, it seems reasonable to hypothesize that habit formation may take longer within an EBPT, as EBPTs often involve establishing and breaking multiple habits. Meanwhile, the parent training EBPT involved 1-h weekly sessions for only 11 weeks, and each new skill was practiced for a mere 7 days. As is true for many EBPTs, this “dosing” seems woefully inadequate to truly form new habits and dismantle old habits. There are several ways EBPTs could promote repetition. At a minimum, key treatment points and skills should become ‘rolling interventions,’ revisited and expanded upon in each session. Brief discussions during each session can be used to assess and encourage progress on the rolling interventions and address obstacles and, very importantly, encourage repetition. Some, but not all, EBPTs already take this approach. In addition, scholars are investigating adding “boosters” (e.g., texts, short sessions) after the main course of treatment has ended (Gearing et al., 2013; Kolko & Lindhiem, 2014). These could promote the repetition of key skills and thus increase the chance of habit formation. Finally, more research is needed to inform the appropriate number of treatment sessions that are needed to promote habit formation. Ideally, this research would evaluate the cost implications of increasing the number of treatment sessions to allow for greater repetition, evaluating whether the resulting improvement in outcomes is substantial enough to justify the expense (Radhakrishnan et al., 2013).

### 2.3. Cues

Habits are formed via the direct association between a stable cue and a behavior. The cues might be internal (e.g., a thought or body sensation) or external (e.g., clock time (Wood & Rünger, 2016). Cues that are salient, accessible and perceptible are the best choices for habit formation (Gardner & Lally, 2018). The parent training EBPT would have benefited from explicit discussion to uncover the cue/s to undesirable parenting habits and to identify and practice using cue/s to prompt the use of new desired parenting habits.

### 2.4. Automaticity

Automaticity arises when we repeat a desired behavior in response to a stable contextual cue. Automaticity is present when a habit is performed with minimal effort or deliberation (Bargh, 1994; Bouton et al., 2011; Verplanken & Orbell, 2003; Wood & Rünger, 2016). This aspect of habits confers multiple advantages because, if our day-to-day routine is engaged in automatically, we are free to devote our attention and energy to more critical aspects of our lives as captured by this famous William James quote: *“The more details of our daily life we can hand over to the effortless custody of automatism, the more our higher powers of mind will be set free for their own proper work”* (James, 1983, p. 34). Unfortunately, I completed the parent training EBPT with very few new parent skills that I engage in automatically. In my opinion, to achieve automaticity, the intervention would need a much more explicit focus on the elements of habit formation discussed here.

### 2.5. Reinforcers

Thorndike’s ‘Law of Effect’ (1927) asserts that behaviors followed by positive outcomes are reinforced and strengthened. Research involving both animals and humans has consistently demonstrated that reinforcers have a substantial impact on the frequency and duration of behaviors (Ferster & Skinner, 1957; Lerner, 2020). The careful assessment, and strategic use, of reinforcers can strengthen habit formation by encouraging consistent repetition of new behaviors in a stable environment. In parent training, parents could be encouraged to recognize when an interaction with one’s child improves as a result of them using the new parenting skills, which is likely to be intrinsically rewarding. Parents could also be encouraged to engage in end of day journaling to provide themselves with positive reinforcement for their efforts.

### 2.6. Current approach

We are at an early stage in a program of research in which are trying to learn about infusing EBPTs with the science of habit formation (Harvey et al., 2022). Here are two examples of our research, both of which are “treatment experiments” aimed at enhancing engagement in healthy sleep-related behaviors.

First, Dr. Laurel Sarfan tested the impact of five habit-change strategies on habits and symptoms among 286 adults with sleep problems. Participants were recruited via Mechanical Turk to change a behavior that is frequently targeted in EBPTs for sleep: wake-up habits (Sarfan et al., 2024). Participants were randomly assigned to a control (i.e., psychoeducation about healthy wake-up habits) or to one of five active habit-change strategies: substitution with RISE UP (Kaplan et al., 2018), awareness training (Azrin & Nunn, 1973), vigilant monitoring (Quinn et al., 2010), implementation intentions (Gollwitzer, 1999), and a values-based approach (Anshel & Kang, 2007). Encouragingly, all five habit-change strategies were associated with improvements in habits and sleep problems, with few differences between conditions. Additionally, change in wake-up habits predicted change in sleep problems, which we interpreted as underscoring the potential clinical utility of targeting habits.

Second, we are currently testing a sleep treatment that is infused with the science of habit formation. We have published a protocol paper that describes the Habits-based Intervention (HABITs) in detail (Diaz et al., 2024). Briefly, one aspect of the approach involves an adaptation of script elicitation, which was developed by Dr. Ben Gardner and colleagues (Mohideen et al., 2023). Script elicitation (a) enables a focus on the elements of habit formation highlighted above and (b) shares similarities with, and can be integrated with, case formulation and functional analysis that are typically central to EBPTs.

There are several steps. First, the therapist collaborates with the patient to identify the aspects of sleep that are most problematic (e.g., getting to sleep, staying asleep, waking up, daytime functioning). Second, we map out the patient’s current routine in the problem domain/s—both the content and the sequencing. In this process, the cues to the behaviors and associated thoughts and emotions are also elicited. In doing so, unhelpful sleep habits are identified. Third, the therapist facilitates the identification of one or more alternative “healthy” habit bundles. A plan is developed for practicing the new habit bundles and dismantling the current unhelpful habitual behaviors. When aiming to dismantle habits, patient’s are encouraged to substitute, curtail, remove or re-organize specific alternatives. To ensure that the alternative habit bundle(s) can be repeated daily, cues are identified to prompt the bundle (s), potential benefits/rewards are identified, and anticipated barriers/obstacles are assessed to support problem solving. Next, in each of 9 weekly sessions, we review progress toward building the new habits and dismantling unwanted habits.

**Fig. 3.**
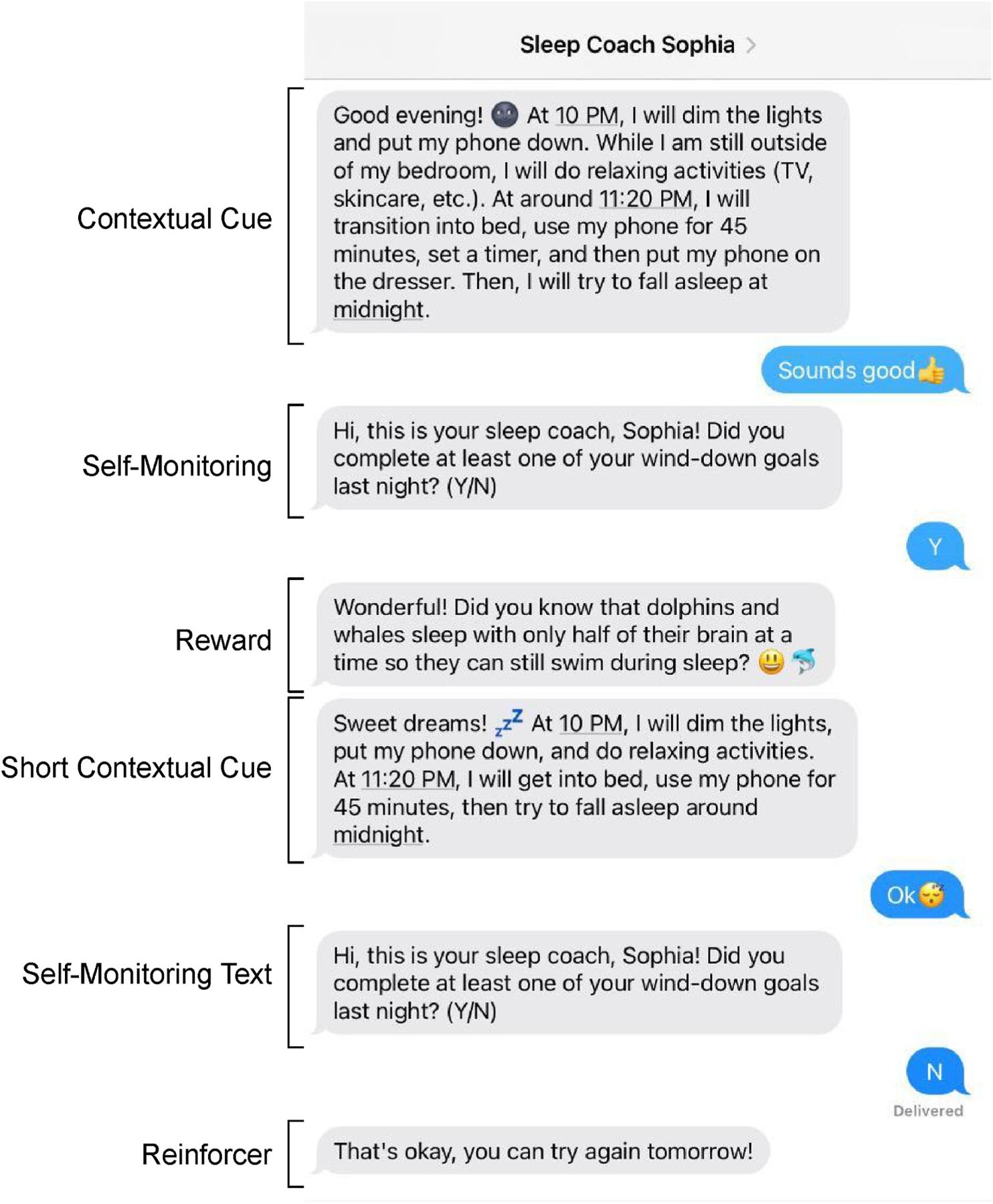
An example of texts designed to promote habit formation (the first 3 texts are sent on the first night, the last 3 texts are sent on the second night).

In addition to receiving the treatment just described, half the participants are randomized into a text messaging intervention. Each text message is personalized to prompt engagement with a primary and secondary habit bundle(s) to be built and dismantled (Diaz et al., 2024). The text message intervention consists of three types of texts: cue texts, self-monitoring texts, and reward texts (see Fig. 3 for an example). The frequency or “dose” of the text messages follows a schedule informed by learning theory (Mowrer, 1960). For reward texts, participants are initially continuously reinforced to rapidly establish a causal relationship between responses and outcomes. This is followed by 4 weeks which are divided between 50% and 33% reinforcement. This switch to partial reinforcement, along with an “expanding-spaced” schedule, was selected to promote resistance to extinction (Nation & Woods, 1980; Tsao & Craske, 2000). The results of this study are eagerly anticipated to determine whether incorporating the science of habit formation into an EBPT proves to be beneficial.

### 2.7. Summary

In sum, infusing the science of habit formation and dismantling into existing EBPTs is proposed as a fruitful domain for future research. There is much to be discovered including: (a) the extent to which current EBPTs succeed in establishing and dismantling habits, (b) if integrating habit formation theory, principles, and measurement into the design and implementation of EBPTs improves outcomes and (c) determining the optimal “dose” of treatment sessions and boosters that are needed for habit formation and disruption.

## 3. Concluding comments

Evidence-based psychological treatments (EBPTs) aim to reverse psychological processes that contribute to the development and/or maintenance of mental illness. Developed and rigorously tested through scientific research, EBPTs effectively address a broad range of mental health challenges, often as front-line treatments (Clark, 2018). There is great potential to continue to maximize outcomes for EBPTs by using basic science findings to develop new adjunctive treatments. Building on Nord et al. (2023), two approaches to maximizing the short and long-term benefits of EBPTs have been described. One aims to improve patient memory for treatment. The other seeks to integrate habit formation science into EBPTs. Adjunctive approaches, such as these, have much potential to boost both short and long-term EBPT outcomes. Yet, significant research is needed in both areas. Thus, we encourage other research teams to explore these and additional approaches to enhancing the effectiveness of EBPTs.

Finally, the research presented herein are examples of the Science of Behavior Change Research Network’s “experimental medicine approach” (Nielsen et al., 2018). This approach involves identifying a specific novel “target” for enhancing EBPTs, such as patient memory for treatment and habit formation. Next, the method for measuring the target must be established. Finally, in a “treatment experiment” the target is experimentally manipulated. Subsequent data analysis includes tests to determine if engaging the target effectively engages the intended mechanism and if this, in turn, improves the outcome. Looping back to the two approaches described in this paper, research on patient memory for treatment has tackled each step of the experimental medicine approach whereas the program of research on habit formation is at the beginning of the process. Grounding research in the experimental medicine approach is valuable as it enables researchers to avoid a “black box” approach in which it is not possible to determine *why* a new treatment or treatment enhancement did or did not work. Also, this approach enables our field to take a “more disciplined scientific approach” as we seek to improve EBPTs (p. 132; Insel, 2009).

## Figures and Tables

**Table 1:** Criteria for selecting memory support strategies from the cognitive psychology and education literatures (Harvey et al., 2014).

The strategy must be precisely defined and operationalizable (identifiable).
The strategy must not overlap or be redundant with other categories, though there may be instances in which a therapist uses multiple strategies at the same time (distinct).
Evidence in the cognitive psychology or education literatures must indicate that this strategy will improve memory (effective).
Therapists can realistically use this strategy within the context of a therapy session (actionable).

**Table 2:** Introduction to the eight memory support strategies (Harvey et al., 2014; Zieve, Dong, et al., 2019).

***Constructive Memory Supports:***
***Cue-based reminders***. Establishing cues that provide reminders increase the potential for transfer of learning (Rogers & Milkman, 2016).
***Categorization*.** There is ample empirical evidence that categorizing information improves recall (Hunt & McDaniel, 1993; Ley et al., 1973) and binding information into meaningful chunks increases memory capacity (Baddeley, 2012; Baddeley & Hitch, 1974).
***Evaluation*.** It is clear that generating and evaluating explanations promotes learning across a wide variety of settings (Graesser et al., 1997; Lombrozo, 2006; Siegler, 2002), and is more effective than spending twice as much time studying (Chi et al., 1994). Evaluation promotes deeper processing (Craik & Lockhart, 1972) as well as conceptual understanding (Murphy & Medin, 1985).
***Application*.** Empirical demonstrations show that people fail to apply learned material to a similar situation that only differs in surface features (Gick & Holyoak, 1983; Lockhart, Lamon, & Gick, 1988). Practicing the application of new knowledge in a variety of contexts assists transfer of learning.
***Non-Constructive Memory Supports:***
***Repetition*.** There is robust evidence that repetition automatizes new knowledge (Guttentag, 1984; Rohrer and Taylor, 2007).
***Practice remembering***. Theories and empirical studies highlight that regenerating, restating and/or rephrasing information improves learning (Ballard, 1913; Karpicke & Roediger, 2007).
***Attention recruitment*.** Theories of memory include a key role for attention (Baddeley, 2012; Baddeley & Hitch, 1974). Experiments show that engaging attention improves memory (Gazzaley & Nobre, 2012; Harrison et al., 2014; Markant & Amso, 2014; Melara et al., 2012).
***Praising recall***. Classic experiments demonstrate that positive consequences for a behavior increases the probability of that behavior (Pavlov, 1927; Skinner, 1938; Thorndike, 1927).

## Data Availability

No data was used for the research described in the article.

## References

[R1] Anshel MH, & Kang M (2007). Effect of an intervention on replacing negative habits with positive routines for improving full engagement at work: A test of the disconnected values model. Consulting Psychology Journal: Practice and Research, 59 (2), 110.

[R2] Azrin N, & Nunn R (1973). Habit reversal: A method of eliminating nervous habits and tics. Behaviour Research and Therapy, 11(4), 619–628. 4777653 10.1016/0005-7967(73)90119-8

[R3] Baddeley A (2012). Working memory: Theories, models, and controversies. Annual Review of Psychology, 63, 1–29. 10.1146/annurev-psych-120710-100422 21961947

[R4] Baddeley AD (2014). Memory theory and memory therapy. In Clinical management of memory problems (2nd edn)(PLE: Memory) (pp. 1–31). Psychology Press.

[R5] Baddeley AD, & Hitch G (1974). Working memory. In Bower GH (Ed.), The psychology of learning and motivation. Academic Press.

[R6] Ballard PB (1913). Oblivescence and reminiscence. British Journal of Psychology Monograph Supplements, 1, 1–82.

[R7] Bargh JA (1994). The four horsemen of automaticity: Awareness, intention, efficiency, and control in social cognition. Handbook of social cognition, 1, 1–40.

[R8] Beck J (1995). Cognitive therapy: Basics and beyond. Guilford.

[R9] Beck JS (2005). Cognitive therapy for challenging problems: What to do when the basics don’t work. Guilford Press.

[R10] Bober SL, Hoke LA, Duda RB, & Tung NM (2007). Recommendation recall and satisfaction after attending breast/ovarian cancer risk counseling. Journal of Genetic Counseling, 16, 755–762. 17674165 10.1007/s10897-007-9109-0

[R11] Bouton ME, Todd TP, Vurbic D, & Winterbauer NE (2011). Renewal after the extinction of free operant behavior. Learning & Behavior, 39(1), 57–67. 21279496 10.3758/s13420-011-0018-6 PMC3059840

[R12] CallawayCA, MilnerAE, ZieveGG, AshlockL, & HarveyAG (2025). The impact of therapist memory for treatment contents on patient outcomes. Manuscript in preparation.10.1016/j.brat.2026.10503741955881

[R13] Callaway CA, Zieve GG, & Harvey AG (2025). The impact of the memory support intervention on therapist memory for treatment contents. Manuscript submitted for publication. 10.1016/j.brat.2026.105104 PMC13711292 42385266

[R14] Chambers MJ (1991). Patient recall of recommendations in the behavioural treatment of insomnia. Sleep Research, 20, 222.

[R15] Chi MTH, de Leeuw N, Chiu MH, & LaVancher C (1994). Eliciting self-explanations improves understanding. Cognitive SCience, 18, 439–477.

[R16] Chi MT, Siler SA, Jeong H, Yamauchi T, & Hausmann RG (2001). Learning from human tutoring. Cognitive science, 25(4), 471–533.

[R17] Chi MT, & Wylie R (2014). The ICAP framework: Linking cognitive engagement to active learning outcomes. Educational Psychologist, 49(4), 219–243.

[R18] Clark DM (2004). Developing new treatments: On the interplay between theories, experimental science and clinical innovation. Behaviour Research and Therapy, 42, 1089–1104. 15325903 10.1016/j.brat.2004.05.002

[R19] Clark DM (2018). Realizing the mass public benefit of evidence-based psychological therapies: The IAPT program. Annual Review of Clinical Psychology, 14, 159–183. 10.1146/annurev-clinpsy-050817-084833 PMC5942544 29350997

[R20] Craik FI, & Lockhart RS (1972). Levels of processing: A framework for memory research. Journal of Verbal Learning and Verbal Behavior, 671–684.

[R21] Croyle RT, Loftus EF, Barger SD, Sun YC, Hart M, & Gettig J (2006). How well do people recall risk factor test results? Accuracy and bias among cholesterol screening participants. Health Psychology, 25, 425–432. 16719615 10.1037/0278-6133.25.3.425

[R22] Diaz M, Ovalle Patino E, Oliver S, Tiab SS, Salazar N, Song J, … Harvey AG (2024). Integrating habit science and learning theory to promote maintenance of behavior change: Does adding text messages to a habit-based sleep health intervention (HABITs) improve outcomes for eveningness chronotype young adults? Study protocol for a randomized controlled trial. Trials, 25(1), 1–21. 39563407 10.1186/s13063-024-08599-4 PMC11577865

[R23] Dong L, Lee JY, & Harvey AG (2017a). Do improved patient recall and the provision of memory support enhance treatment adherence? Journal of Behavior Therapy and Experimental Psychiatry, 54, 219–228. 27614662 10.1016/j.jbtep.2016.08.017 PMC5558150

[R24] Dong L, Lee JY, & Harvey AG (2017b). Memory support strategies and bundles: A pathway to improving cognitive therapy for depression? Journal of Consulting and Clinical Psychology, 85(3), 187. 28221056 10.1037/ccp0000167 PMC5563823

[R25] Dong L, Zieve G, Gumport NB, Armstrong CC, Alvarado-Martinez CG, Martinez A, (2022). Can integrating the memory support intervention into cognitive therapy improve depression outcome? A randomized controlled trial. Behaviour Research and Therapy, 157, Article 104167. 35963181 10.1016/j.brat.2022.104167

[R26] Easterbrook JA (1959). The effect of emotion on cue utilization and the organization of behavior. Psychological Review, 66, 183–201. 13658305 10.1037/h0047707

[R27] Ebbinghaus H (2013). Memory: A contribution to experimental psychology. Ann Neurosci., 20(4), 155–156. 25206041 10.5214/ans.0972.7531.200408 PMC4117135

[R28] Ferster CB, & Skinner BF (1957). Schedules of reinforcement. Appleton-Century-Crofts.

[R29] Fosco WD, Kofler MJ, Groves NB, Chan ES, & Raiker JS (2020). Which ‘working’components of working memory aren’t working in youth with ADHD? Journal of Abnormal Child Psychology, 48, 647–660. 31989344 10.1007/s10802-020-00621-y PMC7192792

[R30] Fournier M, d’Arripe-Longueville F, Rovere C, Easthope CS, Schwabe L, El Methni J, (2017). Effects of circadian cortisol on the development of a health habit. Health Psychology, 36(11), 1059. 28650196 10.1037/hea0000510

[R31] Fournier M, d’Arripe-Longueville F, & Radel R (2017). Testing the effect of text messaging cues to promote physical activity habits: A worksite-based exploratory intervention. Scandinavian Journal of Medicine & Science in Sports, 27(10), 1157–1165. 27540899 10.1111/sms.12730

[R32] Gardner B (2015). A review and analysis of the use of ‘habit’ in understanding, predicting and influencing health-related behaviour. Health Psychology Review, 9(3), 277–295. 25207647 10.1080/17437199.2013.876238 PMC4566897

[R33] Gardner B, Abraham C, Lally P, & de Bruijn G-J (2012). Towards parsimony in habit measurement: Testing the convergent and predictive validity of an automaticity subscale of the Self-Report Habit Index. International Journal of Behavioral Nutrition and Physical Activity, 9(1), 102. 22935297 10.1186/1479-5868-9-102 PMC3552971

[R34] Gardner B, & Lally P (2018). Modelling habit formation and its determinants. In The psychology of habit (pp. 207–229). Springer.

[R35] Gazzaley A, & Nobre AC (2012). Top-down modulation: Bridging selective attention and working memory. Trends in Cognitive Sciences, 16(2), 129–135. 22209601 10.1016/j.tics.2011.11.014 PMC3510782

[R36] Gearing RE, Schwalbe CS, Lee R, & Hoagwood KE (2013). The effectiveness of booster sessions in CBT treatment for child and adolescent mood and anxiety disorders. Depression and Anxiety, 30(9), 800–808. 23596102 10.1002/da.22118

[R37] Ghetti S, & Bunge SA (2012). Neural changes underlying the development of episodic memory during middle childhood. Developmental cognitive neuroscience, 2(4), 381–395. 22770728 10.1016/j.dcn.2012.05.002 PMC3545705

[R38] Gick ML, & Holyoak KJ (1983). Schema induction and analogical transfer. Cognitive Psychology, 15, 1–38.

[R39] Glisky EL (2007). Changes in cognitive function in human aging. Brain aging: Models, methods, and mechanisms, 3–20.

[R40] Gollwitzer PM (1999). Implementation intentions: Strong effects of simple plans. American Psychologist, 54(7), 493.

[R41] Graesser AC, Langston MC, & Baggett WB (1997). Exploring information about concepts by asking questions. In Nakamura GV, Taraban RM, & Medin D (Eds.), Categorization by humans and machines: Vol. 29. The psychology of learning and motivation (pp. 411–436). Academic Press.

[R42] Gumport NB, Dong L, Lee JY, & Harvey AG (2018). Patient learning of treatment contents in cognitive therapy. Journal of Behavior Therapy and Experimental Psychiatry, 58, 51–59. 28869825 10.1016/j.jbtep.2017.08.005 PMC5683909

[R43] Gumport NB, Zieve GG, Dong L, & Harvey AG (2021). The development and validation of the memory support treatment provider checklist. Behavior Therapy, 52 (4), 932–944. 34134832 10.1016/j.beth.2020.11.005 PMC8217732

[R44] Guttentag RE (1984). The mental effort requirement of cumulative rehearsal: A developmental study. Journal of Experimental Child Psychology, 37, 92–106.

[R45] Hahlweg K, & Richter D (2010). Prevention of marital instability and distress. Results of an 11-year longitudinal follow-up study. Behaviour Research and Therapy, 48(5), 377–383. 20053393 10.1016/j.brat.2009.12.010

[R46] Hallford D, Gerring K, Button M, Campbell R, & Sutton K (2024). A randomized controlled component study of cognitive reminiscence therapy for psychological resources and mental well-being: Group-based CRT versus self-guided homework sheets. Cognitive Therapy and Research, 1–16.

[R47] Harrison TL, Mullet HG, Whiffen KN, Ousterhout H, & Einstein GO (2014). Prospective memory: Effects of divided attention on spontaneous retrieval. Memory & Cognition, 42(2), 212–224. 24046252 10.3758/s13421-013-0357-y

[R48] Harvey AG, & Buysse DJ (2017). Treating sleep problems: A transdiagnostic approach. Guilford Publications.

[R49] Harvey AG, Callaway CA, Zieve GG, Gumport NB, & Armstrong CC (2022). Applying the science of Habit Formation to evidence-based psychological treatment: Improving outcomes for mental illness. Perspectives on Psychological Science, 17, 572–589. 10.31234/osf.io/qma4f 34495781 PMC12318445

[R50] Harvey AG, Hein K, Dolsen MR, Dong L, Rabe-Hesketh S, Gumport NB, (2018). Modifying the impact of eveningness chronotype (“Night-Owls”) in youth: A randomized controlled trial. Journal of the American Academy of Child & Adolescent Psychiatry, 57(10), 742–754. 30274649 10.1016/j.jaac.2018.04.020 PMC6923796

[R51] Harvey AG, Lee J, Smith RL, Gumport NB, Hollon SD, Rabe-Hesketh S, (2016). Improving outcome for mental disorders by enhancing memory for treatment. Behaviour Research and Therapy, 81, 35–46. 27089159 10.1016/j.brat.2016.03.007 PMC5559714

[R52] Harvey AG, Lee J, Williams J, Hollon SD, Walker MP, Thompson MA, (2014). Improving outcome of psychosocial treatments by enhancing memory and learning. Perspectives on Psychological Science, 9, 161–179. 25544856 10.1177/1745691614521781 PMC4276345

[R53] Hunt RR, & McDaniel MA (1993). The enigma of organization and distinctiveness. Journal of Memory and Language, 32(4), 421–445.

[R54] Insel TR (2009). Translating scientific opportunity into public health impact: A strategic plan for research on mental illness. Archives of General Psychiatry, 66(2), 128–133. 10.1001/archgenpsychiatry.2008.540 [pii]. 19188534

[R55] James W (1983). Talks to teachers on psychology and to students on some of life’s ideals (Vol. 12). Harvard University Press.

[R56] Jansen J, van Weert J, van der Meulen N, van Dulmen S, Heeren T, & Bensing J (2008). Recall in older cancer patients: Measuring memory for medical information. The Gerontologist, 48(2), 149–157. 18483427 10.1093/geront/48.2.149

[R57] Kaplan KA, Talavera DC, & Harvey AG (2018). Rise and shine: A treatment experiment testing a morning routine to decrease subjective sleep inertia in insomnia and bipolar disorder. Behaviour Research and Therapy, 111, 106–112. 30399503 10.1016/j.brat.2018.10.009

[R58] Karpicke JD, & Roediger HL (2007). Repeated retrieval during learning is the key to long-term retention. Journal of Memory and Language, 57(2), 151–162.

[R59] Kaushal N, & Rhodes RE (2015). Exercise habit formation in new gym members: A longitudinal study. Journal of Behavioral Medicine, 38(4), 652–663. 25851609 10.1007/s10865-015-9640-7

[R60] Kendall PC, & Hedtke KA (2006). Coping cat workbook (child therapy workbooks series). Workbook Publising Company.

[R61] Kolko DJ, & Lindhiem O (2014). Introduction to the special series on booster sessions and long-term maintenance of treatment gains. Journal of Abnormal Child Psychology, 42, 339–342. 24414018 10.1007/s10802-013-9849-2 PMC4011174

[R62] Kravitz RL, Hays RD, Sherbourne CD, DiMatteo MR, Rogers WH, Ordway L, (1993). Recall of recommendations and adherence to advice among patients with chronic medical conditions. Archives of Internal Medicine, 153, 1869–1878. 8250648

[R63] Lally P, Van Jaarsveld CH, Potts HW, & Wardle J (2010). How are habits formed: Modelling habit formation in the real world. European Journal of Social Psychology, 40(6), 998–1009.

[R64] Lee J, & Harvey AG (2015). Memory for therapy in bipolar disorder and comorbid insomnia. Journal of Consulting and Clinical Psychology, 83, 92–102. 25222800 10.1037/a0037911 PMC4323885

[R65] Lee JY, Worrell FC, & Harvey AG (2016). The development and validation of the memory support rating scale. Psychological Assessment, 28(6), 715. 26389597 10.1037/pas0000219 PMC4801757

[R66] Lerner TN (2020). Interfacing behavioral and neural circuit models for habit formation. Journal of Neuroscience Research, 98(6), 1031–1045. 31916623 10.1002/jnr.24581 PMC7183881

[R67] Lewkovich GN, & Haneline MT (2005). Patient recall of the mechanics of cervical spine manipulation. Journal of Manipulative and Physiological Therapeutics, 28, 708–712. 16326241 10.1016/j.jmpt.2005.09.014

[R68] Ley P (1979). Memory for medical information. British Journal of Social & Clinical Psychology, 18, 245–255. 454984 10.1111/j.2044-8260.1979.tb00333.x

[R69] Ley P, Bradshaw P, Eaves D, & Walker C (1973). A method for increasing patients’ recall of information presented by doctors. Psychological Medicine, 3, 217–220. 4715854 10.1017/s0033291700048558

[R70] Ley P, Jain V, & Skilbeck C (1977). A method for decreasing patients’ medication errors. Psychological Medicine, 6(4), 599–601. 10.1017/s0033291700018237 1005577

[R71] Li B, Zhu X, Hou J, Chen T, Wang P, & Li J (2016). Combined cognitive training vs. memory strategy training in healthy older adults. Frontiers in Psychology, 7, 834. 27375521 10.3389/fpsyg.2016.00834 PMC4896109

[R72] Lockhart RS, Lamon M, & Gick ML (1988). Conceptual transfer in simple insight problems. Memory & Cognition, 16, 36–44. 3339997

[R73] Lombrozo T (2006). The structure and function of explanations. Trends in Cognitive Sciences, 10, 464–470. 16942895 10.1016/j.tics.2006.08.004

[R74] Markant J, & Amso D (2014). Leveling the playing field: Attention mitigates the effects of intelligence on memory. Cognition, 131(2), 195–204. 24549142 10.1016/j.cognition.2014.01.006 PMC3963827

[R75] Mcguire LC (1996). Remembering what the doctor said: Organization and adults’ memory for medical information. Experimental Aging Research, 22(4), 403–428. 8968711 10.1080/03610739608254020

[R76] Melara RD, Tong Y, & Rao A (2012). Control of working memory: Effects of attention training on target recognition and distractor salience in an auditory selection task. Brain Research, 1430, 68–77. 22099165 10.1016/j.brainres.2011.10.036

[R77] Menekse M, Stump GS, Krause S, & Chi MT (2013). Differentiated overt learning activities for effective instruction in engineering classrooms. Journal of Engineering Education, 102(3), 346–374.

[R78] Milner AE, Hache RE, Oliver S, Sarfan LD, Spencer JM, Cogan A, (2024). Integrating the memory support intervention into the transdiagnostic intervention for sleep and circadian dysfunction (TranS-C): Can improving memory for treatment in midlife and older adults improve patient outcomes? Study protocol for a randomized controlled trial. Trials, 25(1), 650. 39363383 10.1186/s13063-024-08468-0 PMC11448292

[R79] Mohideen A, Bouvin C, Judah G, Picariello F, & Gardner B (2023). Feasibility and acceptability of a personalised script-elicitation method for improving evening sleep hygiene habits. Health psychology and behavioral medicine, 11(1), Article 2162904. 36618889 10.1080/21642850.2022.2162904 PMC9815428

[R80] Mowrer O (1960). Learning theory and behavior. John Wiley & Sons, Inc.

[R81] Murphy GL, & Medin DL (1985). The role of theories in conceptual coherence. Psychological Review, 92, 289. 4023146

[R82] Nation JR, & Woods DJ (1980). Persistence: The role of partial reinforcement in psychotherapy. Journal of Experimental Psychology: General, 109(2), 175. 7381368

[R83] Nord CL, Longley B, Dercon Q, Phillips V, Funk J, Gormley S, (2023). A transdiagnostic meta-analysis of acute augmentations to psychological therapy. Nature Mental Health, 1(6), 389–401. 38665477 10.1038/s44220-023-00048-6 PMC11041792

[R84] Onken LS, Carroll KM, Shoham V, Cuthbert BN, & Riddle M (2014). Reenvisioning clinical science: Unifying the discipline to improve the public health. Clinical Psychological Science, 2(1), 22–34. 10.1177/2167702613497932 25821658 PMC4374633

[R85] Parikh PK, Troyer AK, Maione AM, & Murphy KJ (2016). The impact of memory change on daily life in normal aging and mild cognitive impairment. The Gerontologist, 56(5), 877–885. 26035897 10.1093/geront/gnv030

[R86] Pavlov IP (1927). Conditioned reflexes. An investigation of the physiological activities of the cerebral cortex. Oxford University Press. 10.5214/ans.0972-7531.1017309 PMC4116985 25205891

[R87] Pickney CS, & Arnason JA (2005). Correlation between patient recall of bone densitometry results and subsequent treatment adherence. Osteoporosis International, 16, 1156–1160. 15744452 10.1007/s00198-004-1818-8

[R88] Quinn JM, Pascoe A, Wood W, & Neal DT (2010). Can’t control yourself? Monitor those bad habits. Personality and Social Psychology Bulletin, 36(4), 499–511. 20363904 10.1177/0146167209360665

[R89] Radhakrishnan M, Hammond G, Jones PB, Watson A, McMillan-Shields F, & Lafortune L (2013). Cost of improving access to psychological therapies (IAPT) programme: An analysis of cost of session, treatment and recovery in selected primary care trusts in the east of england region. Behaviour Research and Therapy, 51 (1), 37–45. 23178677 10.1016/j.brat.2012.10.001

[R90] Rogers T, & Milkman KL (2016). Reminders through association. Psychological Science, 27(7), 973–986. 27207873 10.1177/0956797616643071 PMC5510470

[R91] Rohrer D, & Taylor K (2007). The shuffling of mathematics practice problems improves learning. Instructional Science, 35, 481–498.

[R92] Safren SA, Sprich S, Mimiaga MJ, Surman C, Knouse L, Groves M, (2010). Cognitive behavioral therapy vs relaxation with educational support for medication-treated adults with ADHD and persistent symptoms: A randomized controlled trial. JAMA, 304(8), 875–880. 20736471 10.1001/jama.2010.1192 PMC3641654

[R93] Saleem M, Kühne L, De Santis KK, Christianson L, Brand T, & Busse H (2021). Understanding engagement strategies in digital interventions for mental health promotion: Scoping review. JMIR Mental Health, 8(12), Article e30000. 34931995 10.2196/30000 PMC8726056

[R94] Salthouse TA (2009). When does age-related cognitive decline begin? Neurobiology of Aging, 30(4), 507–514. 19231028 10.1016/j.neurobiolaging.2008.09.023 PMC2683339

[R95] Sarfan LD, Milner AE, Tiab S, Tuli D, & Harvey AG (2024). Let’s kick that habit: A test of five habit-change strategies on habits and symptoms among adults with sleep problems. Manuscript Submitted for Publication. 10.1016/j.jbtep.2025.102049 PMC12385484 40854619

[R96] Sarfan LD, Zieve G, Gumport NB, Xiong M, & Harvey AG (2023). Optimizing outcomes, mechanisms, and recall of Cognitive Therapy for depression: Dose of constructive memory support strategies. Behaviour Research and Therapy, 166, Article 104325. 37210887 10.1016/j.brat.2023.104325 PMC10513748

[R97] Sarfan LD, Zieve GG, Mujir F, Gumport NB, Xiong M, & Harvey AG (2023). Serial mediators of memory support strategies used with Cognitive Therapy for depression: Improving outcomes through patient adherence and treatment skills. Behavior Therapy, 54(1), 141–155. 36608972 10.1016/j.beth.2022.07.012 PMC10927275

[R98] Schacter D (2001). The seven sins of memory: How the mind forgets and remembers. Houghton Mifflin.

[R99] Siegler RS (2002). Microgenetic studies of self-explanations. In Granott N, & Parziale J (Eds.), Microdevelopment: Transition processes in development and learning (pp. 31–58). Cambridge University.

[R100] Skinner BF (1938). The behavior of organisms: An experimental analysis. Appleton-Century-Crofts.

[R101] Stokes TF, & Baer DM (1977). An implicit technology of generalization. Journal of Applied Behavior Analysis, 10(2), 349–367. 16795561 10.1901/jaba.1977.10-349 PMC1311194

[R102] Thew GR (submitted). Treatment Engagement and Between-Session Processes in CBT: Reflections and Future Directions. In Ryum T (Ed.), Facilitating engagement with homework in CBT with complex cases. Springer Nature.

[R103] Thorndike EL (1927). The law of effect. American Journal of Psychology, 39(1/4), 212–222.

[R104] Tsao JC, & Craske MG (2000). Timing of treatment and return of fear: Effects of massed, uniform-, and expanding-spaced exposure schedules. Behavior Therapy, 31 (3), 479–497.

[R105] Tulving E (1989). Remembering and knowing the past. American Scientist, 77(4), 361–367.

[R106] Verplanken B (2018). Introduction. In Verplanken B (Ed.), Psychology of habit (pp. 1–12). Springer.

[R107] Verplanken B, & Orbell S (2003). Reflections on past behavior: A self-Report index of habit Strength 1. Journal of Applied Social Psychology, 33(6), 1313–1330.

[R108] Ward EV, Berry CJ, Shanks DR, Moller PL, & Czsiser E (2020). Aging Predicts decline in explicit and implicit memory: A life-span study. Psychological Science, 31 (9), 1071–1083. 32735485 10.1177/0956797620927648 PMC7521015

[R109] Watkins ER (2018). Rumination-focused cognitive-behavioral therapy for depression. Guilford Publications.

[R110] Weisz JR, Ng MY, & Bearman SK (2014). Odd couple? Reenvisioning the relation between science and practice in the dissemination-implementation era. Clinical Psychological Science, 2(1), 58–74. 10.1177/2167702613501307

[R111] Wood W, & Rünger D (2016). Psychology of habit. Annual Review of Psychology, 67, 289–314. 10.1146/annurev-psych-122414-033417 26361052

[R112] Zayas CE, He Z, Yuan J, Maldonado-Molina M, Hogan W, Modave F, (2016). Examining healthcare utilization patterns of elderly and middle-aged adults in the United States. In The twenty-ninth international flairs conference. PMC4946167 27430035

[R113] Zieve GG, Dong L, Weaver C, Ong SL, & Harvey AG (2019). Patient constructive learning behavior in cognitive therapy: A pathway for improving patient memory for treatment? Behaviour Research and Therapy, 116, 80–89. 30852323 10.1016/j.brat.2019.02.006

[R114] Zieve GG, Gumport NB, Weaver C, McNamara ME, & Harvey AG (2019). Therapist perceptions of client memory for psychological treatment contents and use of memory support strategies: A survey study of clinical practice. Professional Psychology: Research and Practice, 50(5), 288.

[R115] Zieve GG, Sarfan LD, Dong L, Tiab SS, Tran M, & Harvey AG (2023). Cognitive therapy-as-usual versus cognitive therapy plus the memory support intervention for adults with depression: 12-month outcomes and opportunities for improved efficacy in a secondary analysis of a randomized controlled trial. Behaviour Research and Therapy, 170, Article 104419. 37879246 10.1016/j.brat.2023.104419 PMC11025560

[R116] Zieve GG, Woodworth C, & Harvey AG (2020). Client memory and learning of treatment contents: An experimental study of intervention strategies and relationship to outcome in a brief treatment for procrastination. Journal of Behavior Therapy and Experimental Psychiatry, Article 101579. 32459987 10.1016/j.jbtep.2020.101579 PMC7442618

